# From neuronal populations to behavior: a computational journey

**DOI:** 10.3389/fncom.2014.00081

**Published:** 2014-08-01

**Authors:** Arnulf B. A. Graf

**Affiliations:** Division of Biology and Biological Engineering, California Institute of TechnologyPasadena, CA, USA

**Keywords:** neuronal populations, population coding, networks of neurons, decoding, sensory input, behavior

Cognitive behaviors originate in the responses of neuronal populations. We have a reasonable understanding of how the activity of a single neuron can be related to a specific behavior. However, it is still unclear how more complex behaviors are inferred from the responses of neuronal populations. This is a particularly timely problem because multi-neuronal recording techniques have recently become increasingly available, simultaneously spurring advances in the analysis of neuronal population data. These developments are, however, constrained by the challenges of combining theoretical and experimental approaches because both approaches have their unique set of constraints. A solution to this problem is to design computational models that are either derived or inspired by cortical computations.

The focus of this Research Topic is to highlight computational models obtained within the constraints of experimental data collection. The contributors present key findings on subjects including the response properties of single neurons, cortical networks, sensory predictions, and behavioral predictions. Several contributions focused on the question of sensory predictions from neuronal population responses. This topic is essential for understanding the neuronal correlates of behavior, because often it is driven by decisions based on sensory inputs.

We start by studying the response pattern of single neurons. Perona (2014) asked a simple yet elusive question: what if a single Poisson-like neuron was responsible for a binary choice behavior? The author showed that the spike times of the Poisson-like neuron were inherently structured thus making strong predictions for the underlying decision making mechanism. These findings are important for experimental data analysis utilizing the Poisson model, e.g., in the Bayesian inference framework, commonly used for reading out sensory inputs or behaviors based on neuronal population activity.

Next we transition to neuronal populations by investigating the network properties of neurons from a theoretical perspective. Doiron and Litwin-Kumar (2014) derived a global network architecture accounting for the trial-by-trial variability of spike trains when the cortex was either active or at rest. That model accurately predicted experimental findings on interneuronal correlations, a hallmark property of the neuronal population response. Using a different type of network based on the popular Integrate and Fire neurons, Nicola and Campbell (2013) analytically derived mean-field models that they evaluated in numerical simulations using typical parameter values for hippocampal neurons. Using these models, they studied the relation between bursting and adaptation. The findings of these two theoretical studies emphasize the neuronal network properties and their implications on the constraints of computational models.

Next, we investigate how neuronal network properties explain experimental data. The study by (Ba et al., 2014) focused on developing statistical models to analyze the joint spiking activity in neuronal ensembles over small time scale. They designed a multivariate point-process model, and showed how it captured the temporal response properties of thalamic neurons coding the whisker deflection in rats. Models that accurately capture simultaneous events over finer time scales are essential for our understanding of how sensory behavior is represented across neuronal responses. Decoding responses from sensory neurons was also studied by Montijn et al. (2014). In that study the authors recorded from the visual cortex in the mouse to predict visual stimuli based on the neuronal population response. The aim of the study was to understand whether and how interneuronal correlations and the shape of individual neuronal tuning curves affected the decoding accuracy. The authors found that both factors impacted the decoding accuracy. Similarly, the importance of interneuronal correlations for predicting sensory inputs and behaviors was further highlighted by Snyder et al. (2013) in primate visual cortex. They focused on understanding the dynamics of behavioral and sensory processing across neuronal populations by analyzing correlations on trial-by-trial basis. The authors derived a complementary metric to interneuronal correlations that used the variability across the population response. This study provided computational models, validated them with experimental data, and showed how to optimally combine the responses across neuronal populations to analyze cortical representations over different time scales.

We conclude this Research Topic by looking at computational models of a complex cognitive behavior: attention. Attention is known to modulate the response properties of single neurons in various ways and computational models can account for the diversity of effects. However, it is less clear how to extend the predictions of these models to responses of neuronal populations, which tend to be heterogeneous. The paper by Hara et al. (2014) addressed this question for the normalization model of attention and showed that the predictions of the model varied drastically for single neurons and neuronal population. They provided a practical guide on how to extend the model to account for heterogeneity in neuronal populations, essential for understanding the complexity of cognitive behaviors.

The contributions to this Research Topic examined how behaviors are *encoded* in the responses of neuronal populations. The authors showed us how to create encoding models that describe the data by making empirically driven assumptions, and how to model the putative mechanisms that optimally combine the responses from multiple neurons. The contributors then built on these findings, and demonstrated how to compute *decoding* models that predict either sensory inputs or behavioral outcomes corresponding to given neuronal population activities. Taken together, the findings presented in this Research Topic have shown repeatedly that decoding from neuronal populations is more than merely the sum of its parts: novel features, hidden at the level of single neurons, arise when pooling neurons together. To understand the properties of such novel features, we need to develop a systematic methodology by approaching this problem from multiple levels ranging from theoretical modeling to empirical validations. This Research Topic shows that empirically constrained decoding schemes are essential for our understanding of how the activity of neuronal populations represents behavior.

## Conflict of interest statement

The author declares that the research was conducted in the absence of any commercial or financial relationships that could be construed as a potential conflict of interest.
